# Strengthening family medicine: a Canadian perspective and the RCGP International and Overseas Network

**DOI:** 10.3399/bjgpopen17X101301

**Published:** 2017-12-13

**Authors:** Gina Agarwal

**Affiliations:** 1 Associate Professor, Department of Family Medicine, McMaster University, Hamilton, Ontario, Canada; 2 Founder Member, RCGP International and Overseas Network, RCGP, London, UK

**Keywords:** Family medicine, General practice, Canada, International and Overseas Network, Royal College of General Practitioners

Canada is a relatively young country and therefore its system of family medicine has relied heavily on the medicine practiced in other similar nations, particularly the UK. However, Canada has developed a set of 'Four Principles of Family Medicine'^1^ that would be relevant to GPs all over the globe. Canadian family physicians are taught that:

‘... the family physician is a skilled clinician; family medicine is a community-based discipline; the family physician is a resource to a defined practice population; the patient–physician relationship is central to the role of the family physician’.

Sounds familiar, doesn’t it? Principles that resound whatever country one practices in! The learning to be gained from these principles, wherever they are practised, is something that the Royal College of General Practitioners (RCGP) International and Overseas Network (ION) members could strive towards.

## The Canadian system of family medicine

In Canada, family physicians complete a family medicine residency programme at an accredited university postgraduate programme, such as McMaster University’s Department of Family Medicine Programme. This occurs after they obtain a medical degree. Residency takes 2 years to complete and is based around the ‘Triple C curriculum’ in family medicine.^2^ The three components of Triple C are: *comprehensive* education and patient care, and *continuity* of education and patient care, *centred* in family medicine. The curriculum is competency-based, using carefully designed curricular elements to achieve pre-stated desired outcomes. Material has been developed by the College of Family Physicians of Canada (CFPC) to assist residency programmes in identifying appropriate educational opportunities for their residents. Residents track and document the achievement of these competencies throughout their residency. To obtain these skills, residents will attend hospital and community practices, with an emphasis on being able to complete the certification exam from the CFPC. Canadian family physicians take on ‘cradle-to-grave’ care of their patients, although practice will differ in rural and urban areas. Rural practices will be more comprehensive, and may include emergency medicine, obstetrics, anaesthetics, or other areas of specialisation.

## Big issues affecting health care and family medicine in Canada

There are some systemic issues and some more clinical issues that are paramount in Canadian family practice currently. Healthcare system issues concern what the optimal models for primary care provision might be, and how they should be remunerated. Although capitation payment systems have existed for a very long time in the UK, they are newer in Canada, and the older system of fee-for-service is still in evidence. Formation of large, interdisciplinary teams to care for groups of patients is also a cutting-edge phenomenon. They may contain nurse practitioners, physician assistants, community paramedics, and other allied health staff. The concept of the walk-in clinic, for people to bypass their family doctor and get immediate and fast primary care, is somewhat controversial and political. Access to care and, in particular, differences in access to care between urban and rural areas are very much at the forefront of decisions that are made in health policies affecting primary care. Clinical issues in Canadian primary care that are all over the news include the legalisation of cannabis, and how family physicians will be involved in this and what the implications might be. Medical assistance in dying is also being discussed, as patients are making their wishes known, since recent legislation has legalised euthanasia across all Canadian provinces.

## What and how can we learn from each other

Although both Canada and the UK are high income countries with many similarities, there is much to be learnt. Canadians have a well-developed continuing medical education system, including problem-based learning groups and other small-group education, that has been very well received and successful. Problem-based learning, as a method, originated in Canada,^3^ and has spread to institutions around the world. However, Canadian family practice could learn mutually from the UK consultation models that are taught in vocational training.

The ION aims to assist in the development of collaborative learning and projects between the UK and countries in which the RCGP’s International and Overseas members practice. Canada has a fair number of Overseas MRCGP-holders. This network of people could be utilised to spread UK-based general practice methods to Canadian family medicine residents who, in turn, could impart their knowledge to UK GP trainees. It is a concept not yet capitalised on, but one that the ION hopes to take on, and is just one of the myriad of ideas that its founder members are bursting with.
